# Antifungal Effect of Henna against *Candida albicans* Adhered to Acrylic Resin as a Possible Method for Prevention of Denture Stomatitis

**DOI:** 10.3390/ijerph13050520

**Published:** 2016-05-23

**Authors:** Amal Nawasrah, Amani AlNimr, Aiman A. Ali

**Affiliations:** 1 Departments of Substitutive Dental Sciences, College of Dentistry, University of Dammam, Dammam 31555, Saudi Arabia; amnawasrah@gmail.com; 2 Clinical Microbiology, Department of Medical Microbiology, College of Medicine, University of Dammam, Dammam 31555, Saudi Arabia; amalnimr@uod.edu.sa; 3 Oral Pathology and Medicine, Department of Biomedical Dental Sciences, College of Dentistry, University of Dammam, PO Box 60710, Dammam 31555, Saudi Arabia

**Keywords:** henna, denture stomatitis, acrylic denture resin

## Abstract

Denture stomatitis is a very common disease affecting the oral mucosa of denture wearers. The aim of this study was to measure the antifungal effect of henna against *Candida albicans* adhered to acrylic resin as a possible method for prevention of denture stomatitis. One-hundred-eighty acrylic plates were prepared of heat-cured acrylic denture resin. The specimens were divided into six groups of 30 samples each. The first group was only polymer and monomer following the conventional manufacturer instruction for processing complete dentures. The other five groups were processed by adding different concentration of Yamani henna powder (Harazi) to the polymer in a concentration of henna: polymer 1%, 2.5%, 5%, 7.5% and 10%, respectively. Samples were incubated in artificial saliva rich with *Candida albicans* at 37 °C, and the effect of henna on *Candida albicans* was evaluated in two different methods: semi-quantitative slide count and a culture-based quantitative assay (quantitative). Variation in the number of live Candida was observed with the increase in the concentration of Yamani henna powder. It was observed that the variation in live *Candida*, between control group and group B (concentration of Yamani henna powder was 1%), was statistically significant with a *p*-value of 0.0001. Similarly, variations in live *Candida* were significant, when the concentration of powder was 7.5% or 10% in contrast with control group and *p*-values were 0.0001 and 0.001 respectively. Adding henna to acrylic resin denture could be effective in controlling *Candida albicans* proliferation on the denture surface; however, its effects on the physical properties of acrylic resin denture need further studies.

## 1. Introduction

One of the difficulties encountered during dental care is the treatment of denture stomatitis (DS). This prevalent and longstanding problem is common in old people wearing complete or partial dentures [1], and it is related mainly to the adhesion of *Candida albicans* to the rough denture surface [1,2,3,4,5]. The prevalence of DS was high in denture users [6]. A review reported DS in 11%–67% of complete denture wearers. It is more common on the palatal mucosa [7], and affects females more [1,2].

The material used to construct the denture is acrylic resin that has acceptable physical, mechanical and esthetic properties with the defect of allowing the adhesion of microbes [8,9]. The adhesion of microbes to the acrylic resin has many factors including surface properties of acrylic resin [10].

As DS is a fungal disease, many investigators have suggested different antifungal modalities for treating it [1,11,12]. Douglas and Walker in 1973 [11] tested the efficacy of denture lining materials containing antifungals in the treatment of DS, confirmed the inhibitory effect of tissue conditioners incorporated with nystatin. While Thomas and Nut in 1978 [12] found that the addition of nystatin powder to tissue conditioner was successful in inhibiting the growth of *Candida albicans*, *Candida tropicalis*, and *Candida krusei*. Kuhn in 2002 [13] mentioned that the problem encountered with treating *Candida albicans* is enhancing resistance to antimicrobials and decreasing susceptibility to a variety of antifungal agents (including fluconazole, nystatin, chlorhexidine, terbenafine, amphotericinB, and the triazolesvoriconazole and ravuconazole) [14].

Many other investigators tried to manage this problem by controlling the adhesion of *Candida albicans* on the acrylic resin surface [15,16,17]. Some studies showed that coating the acrylic resin surface with 2 oktyl cyanoacrylate [15], silane-SiO_2_ nanocomposite films [16] or experimental coatings containing zwitterion or hydrophilic monomers [17] will prevent the adhesion of *Candida albicans* on the acrylic resin surface.

Henna is a natural product [18] that is cheap, and easy to find and apply by the patient, with an approved antifungal effect. Utilizing henna to treat and even prevent the adherence of *Candida albicans* to acrylic resin is a valuable study to establish. When henna is mixed with water, it forms a paste that can be applied directly to the skin. Darker skin tones can be obtained with longer durations of contact [19]. Henna has been used for a long time to color skin and hair by applying it to skin for several hours to be absorbed; this way will result in a brownish orange stain [18].

Henna is safe without any health effects [20]. The side effect of henna mainly includes allergic reactions, but few have been reported. Kirkland and Marzin [21,22] declared that henna has no genotoxic risk to the consumers, and this fact was also confirmed by Yusufetal [18].

A phytopharmacological review in 2010 reported that henna has analgesic, hypoglycemic, hepatoprotective, immunostimulant, anti-inflammatory, antibacterial, antimicrobial, antifungal, antiviral, antiparasitic, antitrypanosomal, antidermatophytic, antioxidant, antifertility, tuberculostatic and anticancer properties [23]. Henna is found to have other pharmacological uses such as antitumor [24], anthelmintic [25], antioxidant, immunomodulatory [26], burn wound healing [27], UV protective [28] and antimicrobial properties [29].

Moreover, other studies declared that henna leaves have anti-microbial and anti-*Candida albicans* effect [29,30].

The minimum inhibitory concentration (MIC) is defined as the lowest concentration of an agent that causes inhibition of visible growth of a tested microorganism. Phadungcharoen *et al.* reported that the minimal amount of Lawsone methyl ether mouthwash required for inhibiting the growth of *Candida albicans* was 0.4 g/L (to check this, the MIC unit needs a volume e.g., g/L or g/mL, *etc.*) [31]. Other studies found that henna is active against non-dermatophyte fungi (*Aspergillus niger*, *Candida albicans* and *Cryptococcus* sp.) with a MIC range of 0.75–25 g/mL [32].

So far, no study has been undertaken to investigate the antifungal properties of henna on denture stomatitis, so this study aims to evaluate dyeing characteristics and antifungal efficacy of henna powder processed with acrylic resin against fungus *Candida albicans*. We quantified the fungicidal effect of henna powder to assess its potential in preventing *Candida albicans* adherence to acrylic resin as a possible method for prevention of denture stomatitis.

## 2. Materials and Methods

### 2.1. Sample Size and Specimen Preparation

One-hundred-eighty acrylic plates were prepared of heat cured acrylic (Trevalon/Universal Clear–DENTSPLY, Konstanz, Germany), with a powder and liquid ratio, similar to the ones made for complete dentures. The specimens were divided into 6 groups of 30 samples each. The first group was only polymer and monomer following the conventional manufacturer instruction for processing complete denture. The other five groups were processed by adding different concentration of Yamani henna powder (Harazi, Sanaa, Yemen) to the polymer in a concentration of henna: polymer 1%, 2.5%, 5%, 7.5% and 10% respectively. Samples were fabricated at the prosthodontic laboratory in the College of Dentistry, University of Dammam, with dimensions of 20 × 10 × 3 mm.

### 2.2. Exposing Acrylic Specimens to Candida albicans

All acrylic plates were immersed in artificial saliva that contains 2,000,000 cells of *Candida albicans* (ATCC 10231) and incubated in 37 °C for 2 weeks. All of the samples were then washed with tap water and subjected for evaluation of the number of *Candida albicans* attached and proliferated on the surface of acrylic resin samples.

### 2.3. Evaluation

Two methods were used to calculate the number of live *Candida albicans* adherent to each acrylic resin sample as follows: after washing each plate with tap water, the plates were incubated in a broth at 37 °C for 48 h, then vibrated using a vortex followed by centrifuging the tubes with specimens to get the concentrated bullet of *Candida*. Then, the following 2 methods of counting were used for each specimen:

1- Slide count: 2.5 µL of Trypan Blue 0.4% solution in Phosphate (MP-Biomedicals, Santa Ana, CA, USA) was added to 7.5 µL of each sample to be placed on a slide count (Nebauer Slide Counter “Chambers-Marienfeld”, Marienfeld, Lauda-Königshofen, Germany) for microscopic evaluation. With Trypan Blue stain, dead *Candida albicans* would appear blue in color while live *Candida* would appear transparent with a blue border line. Trypan Blue stain should allow for counting the number of *Candida* under light microscope at low power magnifications (10×). Slide count usually contains 4 main squares, and each is divided into 16 squares. *Candida* was counted in 2 main squares and multiplied by 2 to get the total number of *Candida* in the slide.
2- Miles and Misra assay: This is the standard, accurate culture-based test to quantify live organisms. Briefly, 10 µL of each sample was taken, serially diluted and spread on a quadrant of a Petri dish and then it was incubated at 37 °C for 48 h. Colonies of *Candida albicans* were counted, using a marker pen counter (colony counter “Scienceware-bel-art products”, Wayne, NJ, USA), in the quadrant where acceptable growth is noted and corrected for the dilution factor. If the number of colonies that cover the whole surface of the Petri dish was more than 500, it was considered as overgrowth.
3- Statistics: Because the discrete colony count values varied a lot, the mode method was used to represent the results. Log reduction of each concentration by both methods was reported with *p*-value calculated by the paired *t*-test to check for robustness. To compare the performance of the two assays, the Bland–Altman plot was performed [33] using Graph Pad 6.0 for Windows (GraphPad Software, La Jolla, CA, USA) to illustrate method agreement. It plotted the difference in average counts between the two methods. All results for all concentration and growth control were included in the analysis. The software calculated the bias that represents the average discrepancy between the methods, and the upper and lower levels of agreement (LOA) calculated as bias ±1.96 X standard deviation of the bias. The closer the bias is to zero and the narrower the limits of agreement, the more agreed the methods are.

## 3. Results

### 3.1. Slide Count

Live *Candida albicans* were counted for group A (Control) to group F by using the slide count method. Variation in the number of live *Candida* was observed with the increase in the concentration of Yamani henna powder (Harazi) (Table 1). It was observed that the variation in live *Candida*, between control group and group B (concentration of Yamani henna powder was 1%), was statistically significant with a *p*-value of 0.0001. Similarly, variations in live *Candida* were significant, when the concentration of powder was 7.5% or 10% in contrast with control group, and *p*-values were 0.0001 and 0.001, respectively.

### 3.2. Direct Culture Method

**Figure 1:**
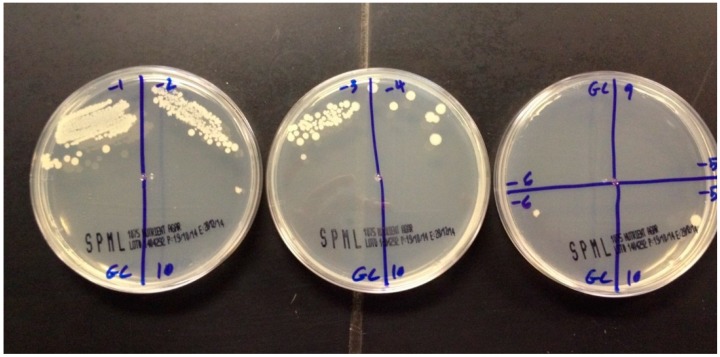
Different cultures showing different concentrations of *Candida* colonies.

The number of live *Candida* was also checked by a culture test (Figure 1), and mean and standard deviation was obtained for each group. Variations of live *Candida* for each group in comparison with the control group was checked for statistical significance. ANOVA was employed and found that when the concentration of henna powder was 1%, 10% of the results were significant with *p*-values 0.001 and 0.01, respectively.

Statistical difference, in the mean number of live *Candida* by using two different methods, was also evaluated. Significant difference in mean was only observed when the concentration of henna powder was 1% and 2.5% with the *p*-values 0.002 and 0.001, respectively.

**Figure 2:**
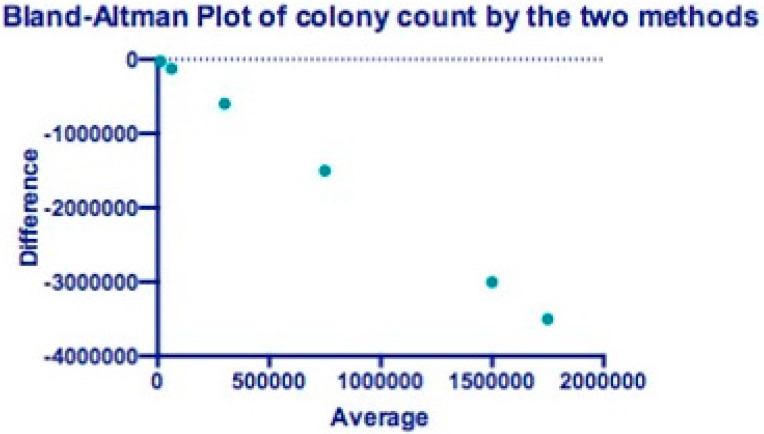
Bland–Altman Plot of colony count by two methods.

The mean difference in the number of live *Candida albicans* between the two methods (slide count and direct culture test) was −140,757,122 and difference between the means, obtained from two different methods, was statistically significant with *p*-value of 0.00025. Ninety-five percent of confidence limits were laid between −1,054,423,691 and 772,909,448 (Figure 2).

The Bland–Altman Plot in Figure 1 showed the differences in average *Candida* colony counts in two methods. The bias is the average difference—the closer to zero, the more concordant the method. The upper and lower limits of agreement (LOA) represent bias ±1.5 SD of the bias. The upper and lower LOA for the agar dilution method and counting chamber in this experiment are (−4.4, 1.5).

## 4. Discussion

The purpose of this study was to measure the antifungal effect of henna against *Candida albicans* adhered to acrylic resin as a possible method to treat and prevent denture stomatitis for complete denture wearers. It was found that henna powder could have an antifungal effect on the acrylic resin, the material of the complete denture.

The only significant difference in reducing the number of *Candida albicans*, considered the most pathogenic factor causing denture stomatitis [15,34], was with 1% and 10% henna using both methods of tests. The results indicated that henna can be effective in producing antimicrobial environment against *Candida albicans*.

Many studies found that henna has an antifungal effect and could be used to treat fungal infection as a substitute to the drugs [34]. Some studies showed the antimicrobial effect on the acrylic resin by treating the surface of the denture with certain materials in order to reduce the chance of *Candida* adherent [15,34]. This idea is derived from considering the surface of the acrylic resin as an area of plaque adherence and colonization [35]. It was also suggested that hydrophilic material coating may be effective in reducing the adherence of *Candida albicans*. [36]. Other studies dealt with adding the antimicrobial material to the denture base to formulate what they called potential antimicrobial denture base resin, but concern about the biocompatibility and the concentration needed without affecting the physical properties is still there [37]. Thus, the focus for modifying the denture base made of poly methyl methacrylate (PPMA) was either modifying the surface or through chemical modification [38].

Our research dealt with the second type of modification, but it was different using henna as a natural product from plant added to acrylic powder, mixed with the monomer and processed.

Since henna is cheap, available and has an antifungal effect on acrylic resin, the processing method of the complete denture does not add more machines or complicated steps when using henna; this type of research can be significant in preventing the adherence of *Candida albicans* that is related to the development of denture stomatitis. The drugs used to treat denture stomatitis need at least two weeks to be efficient. Furthermore, the infection will be back after a short period of drug cessation [39].

As shown in Table 1, initial screening has shown that henna powder can be potential antifungal compounds to be developed further with log reduction ranging between 6 and 6.7 in both methods used. However various efficacies were shown in different concentrations as tested by each method. Of note, at 5% henna, the effect was suboptimal, even lower than that of lower concentrations of the agent (1% and 2.5%). This can be explained by either the coincidence of several tubes showing no growth of *Candida* at all, which might have reduced the accuracy of the overall estimate of colony counts at this concentration as a random error, or it could be due to factual reduction of henna activity at this level due to kinetics considerations that are difficult to explain in the early screening of a compound. Some antimicrobials are known to require a particular concentration ratio with the organism. Relevant pharmacokinetics data are important in order to test this hypothesis in future experimental work.

When screening a novel product, accurate but non-labor intensive techniques are desired. In this study, the counting chamber method performed well in concordance with the reference agar dilution method and gave similar log reductions with robustness and good agreement in the Bland-Altman plot (Figure 2). Although similar in principle to the agar dilution, its advantages include practicality, ease and speed compared to the agar dilution method. Although our study reports that adding henna to acrylic resin dentures will inhibit the proliferation of *Candida albicans*, studying the effect of adding henna on the physical and chemical properties of acrylic resin dentures is required.

## 5. Conclusions

In conclusion, adding henna to the acrylic resin has shown, by two different techniques in this study, that it can control *Candida* proliferation. These initial screening results are encouraging for further investigation of henna compounds, especially in regards to their toxicity and pharmacokinetic properties. Future work should consider testing these lead compounds against *in vitro* biofilm models. In addition, testing the compounds against various strains will be clinically informative.

## Figures and Tables

**Table 1:** Effect of different concentrations of henna on *Candida albicans*.

Group	Agar Dilution Mode	Log Reduction in Candida	*p*-Value	Counting Chamber Mode	Log Reduction in Candida	*p*-Value
A	5 × 10^6^	-	-	2 × 10^6^	-	-
B 1%	2 × 10^6^	6.5	0.0001 *	1 × 10^6^	6	0.001 *
C 2.5%	5 × 10^5^	6.7	0.2	7 × 10^5^	6.1	0.1
D 5%	4 × 10^6^	6	0.6667	2 × 10^6^	NA	NA
E 7.5%	1 × 10^5^	6.7	0.0001 *	1.5 × 10^5^	6.3	0.1
F 10%	1 × 10^4^	6.7	0.001 *	4 × 10^4^	6.3	0.01 *

* Statistically significant at 0.05 level of significance.

## References

[1] Pattanaik S. (2010). Denture Stomatitis: A Literature Review. J. Indian Acad. Oral Med. Radiol..

[2] Figueiral M.H., Azul A., Pinto E., Fonseca P.A., Branco F.M., Scully C. (2007). Denture-related stomatitis: Identification of aetiological and predisposing factors—A large cohort. J. Oral Rehabil..

[3] Zomorodian K., Haghighi N.N., Rajaee N., Pakshir K., Tarazooie B., Vojdani M., Sedaghat F., Vosoghi M. (2011). Assessment of Candida species colonization and denture-related stomatitis in complete denture wearers. Med. Mycol..

[4] Pires F.R., Santos E.B.D., Bonan P.R.F., De Almeida O.P., Lopes M.A. (2002). Denture stomatitis and salivary Candida in Brazilian edentulous patients. J. Oral Rehabil..

[5] Ramage G., Tomsett K., Wickes B.L., López-Ribot J.L., Redding S.W. (2004). Denture stomatitis: A role for Candida biofilms. Oral Surg. Oral Med. Oral Pathol. Oral Radiol. Endod..

[6] Kossioni A.E. (2011). The prevalence of denture stomatitis and its predisposing conditions in an older Greek population. Gerodontology.

[7] Arendorf T.M. (1987). Denture stomatitis: A review. J. Oral Rehabil..

[8] Jain D., Shakya P. (2013). An *in vitro* study on effect of Delmopinol application on *Candida albicans* adherence on heat cured denture base acrylic resin: A thorough study. Indian J. Dent. Res..

[9] Castro D.T.D., Holtz R.D., Alves O.L., Watanabe E., Valente M.L.D.C., Silva C.H.L.D. (2014). Development of a novel resin with antimicrobial properties for dental application. J. Appl. Oral Sci..

[10] Pereira-Cenci T., Del Bel Cury A.A., Crielaard W., Ten Cate J.M. (2008). Development of Candida-associated denture stomatitis: New insights. J. Appl. Oral Sci..

[11] Douglas W., Walker D. (1973). Nystatin in denture liners—An alternative treatment of denture stomatitis. Br. Dent. J..

[12] Thomas C., Nutt G. (1978). The *in vitro* fungicidal properties of Visco-gel, alone and combined with nystatin and amphotericin B. J. Oral Rehabil..

[13] Kuhn D., George T., Chandra J., Mukherjee P., Ghannoum M. (2002). Antifungal susceptibility of Candida biofilms: Unique efficacy of amphotericin B lipid formulations and echinocandins. Antimicrob. Agents Chemother..

[14] Coogan M.M. (2006). (B1) Candida and mycotic infections. Adv. Dent. Res..

[15] Ali A.A., Alharbi F.A., Suresh C.S. (2013). Effectiveness of coating acrylic resin dentures on the *Candida adhesion*. J. Prosthodont..

[16] Yodmongkol S., Chantarachindawong R., Thaweboon S., Thaweboon B., Amornsakchai T., Srikhirin T. (2014). The effects of silane-SiO_2_ nanocomposite films on *Candida albicans* adhesion and the surface and physical properties of acrylic resin denture base material. J. Prosthet. Dent..

[17] Izumida F.E., Moffa E.B., Vergani C.E., Machado A.L., Jorge J.H., Giampaolo E.T. (2014). *In vitro* evaluation of adherence of *Candida albicans*, *Candida glabrata*, and *Streptococcus mutans* to an acrylic resin modified by experimental coatings. Biofouling.

[18] Yusuf M., Ahmad A., Shahid M., Khan M.I., Khan S.A., Manzoor N., Mohammad F. (2012). Assessment of colorimetric, antibacterial and antifungal properties of woollen yarn dyed with the extract of the leaves of henna (*Lawsonia inermis*). J. Clean. Prod..

[19] Ramírez-Andreo A., Hernández-Gil A., Brufau C., Marín N., Jiménez N., Hernández-Gil J., Tercedor J., Soria C. (2007). Allergic contact dermatitis to temporary Henna tattoos. Actas Dermo-Sifiliogr. (Engl. Ed.).

[20] Bele A.A., Jandhav V.M., Kadam V.J. (2010). Potential of Tannnins: A review. Asian J. Plant Sci..

[21] Kirkland D., Marzin D. (2003). An assessment of the genotoxicity of 2-hydroxy-1, 4-naphthoquinone, the natural dye ingredient of Henna. Mutat. Res. Genet. Toxicol. Environ. Mutagen..

[22] Marzin D., Kirkland D. (2004). 2-Hydroxy-1, 4-naphthoquinone, the natural dye of Henna, is non-genotoxic in the mouse bone marrow micronucleus test and does not produce oxidative DNA damage in Chinese hamster ovary cells. Mutat. Res. Genet. Toxicol. Environ. Mutagen..

[23] Chaudhary G., Goyal S., Poonia P. (2010). *Lawsonia inermis* Linnaeus: A Phytopharmacological Review. Int. J. Pharm. Sci. Drug Res..

[24] Jha R.K., Jha P.K., Rana S.V., Guha S.K. (2009). Spermicidal action of styrene maleic anhydride polyelectrolyte in combination with magnetic and electrically conductive particles. Int. J. Pharmacol..

[25] Bairagi G., Kabra A., Mandade R. (2011). Anthelmintic activity of *Lawsonia inermis* L. leaves in Indian adult earthworm. Int. J. Res. Pharm. Biomed. Sci..

[26] Mikhaeil B.R., Badria F.A., Maatooq G.T., Amer M. (2004). Antioxidant and immunomodulatory constituents of henna leaves. Z. Naturforschung C.

[27] Muhammad H., Muhammad S. (2005). The use of *Lawsonia inermis* Linn. (Henna) in the management of burn wound infections. Afr. J. Biotechnol..

[28] Dweck A.C. (2002). Natural ingredients for colouring and styling. Int. J. Cosmet. Sci..

[29] Saadabi M.A.A. (2007). Evaluation of *Lawsonia inermis* Linn. (Sudanese Henna) leaf extracts as an antimicrobial agent. Res. J. Biol. Sci..

[30] Abulyazid I., Mahdy E.M.E., Ahmed R.M. (2013). Biochemical study for the effect of henna (*Lawsonia inermis*) on *Escherichia coli*. Arabian J. Chem..

[31] Sritrairat N., Nukul N., Inthasame P., Sansuk A., Prasirt J., Leewatthanakorn T., Piamsawad U., Dejrudee A., Panichayupakaranant P., Pangsomboon K. (2011). Antifungal activity of lawsone methyl ether in comparison with chlorhexidine. J. Oral Pathol. Med..

[32] Ponnusamy K., Petchiammal C., Mohankumar R., Hopper W. (2010). *In vitro* antifungal activity of indirubin isolated from a South Indian ethnomedicinal plant *Wrightia tinctoria* R. Br.. J. Ethnopharmacol..

[33] Dewitte K., Fierens C., Stöckl D., Thienpont L.M. (2002). Application of the Bland-Altman plot for interpretation of method-comparison studies: A critical investigation of its practice. Clin. Chem..

[34] Singla S., Gupta R., Puri A., Singh V., Roy S. (2013). Comparison of antiCandidal activity of *Punica granatum* (Pomegranate) and *Lawsonia inermis* (Henna leaves): An *in vitro* study. Int. J. Dent. Res..

[35] Gendreau L., Loewy Z.G. (2011). Epidemiology and etiology of denture stomatitis. J. Prosthodont..

[36] Yoshijima Y., Murakami K., Kayama S., Liu D., Hirota K., Ichikawa T., Miyake Y. (2009). Effect of substrate surface hydrophobicity on the adherence of yeast and hyphal Candida. Mycoses.

[37] Sivakumar I., Arunachalam K.S., Sajjan S., Ramaraju A.V., Rao B., Kamaraj B. (2014). Incorporation of antimicrobial macromolecules in acrylic denture base resins: A research composition and update. J. Prosthodont..

[38] Raj P.A., Dentino A.R. (2013). Denture polymers with antimicrobial properties: A review of the development and current status of anionic poly (methyl methacrylate) polymers. Future Med. Chem..

[39] Chandra J., Mukherjeel P.K., Leidichl S.D., Faddoul F.F., Hoyer L.L., Douglas L.J., Ghannouml M.A. (2001). Antifungal resistance of Candidal biofilms formed on denture acrylic *in vitro*. J. Dent. Res..

